# Urinary and serum biomarkers of renal injury in coronary artery bypass grafting: a prospective evaluation with new biomarkers’

**DOI:** 10.1590/2175-8239-JBN-2024-0173en

**Published:** 2025-07-18

**Authors:** Antônio Felipe Leite Simão, Gdayllon Cavalcante Meneses, Lia Cavalcante Cezar, Letícia Machado de Araújo, Alice Maria Costa Martins, Heraldo Guedis Lobo, Bruna Viana Barroso Martins, Geraldo Bezerra da Silva, Elizabeth De Francesco Daher, José Glauco Lobo

**Affiliations:** 1 Universidade Federal do Ceará, Hospital Universitário Walter Cantidio, Fortaleza, CE, Brazil.; 2 Universidade Federal do Ceará, Faculdade de Medicina, Departamento de Fisiologia e Farmacologia, Programa de Pós-Graduação em Ciências Médicas, Fortaleza, CE, Brazil.; 3 Universidade Federal do Ceará, Faculdade de Medicina, Departamento de Cirurgia, Programa de Pós-Graduação em Ciências Médicas, Fortaleza, CE, Brazil.; 4 Hospital de Messejana Dr. Carlos Alberto Studart Gomes, Fortaleza, CE, Brazil.; 5 Xiamen University, Xiamen Cardiovascular Hospital, Xiamen, China.; 6 Universidade Federal do Ceará, Faculdade de Medicina, Departamento de Imunologia, Fortaleza, CE, Brazil.; 7 Universidade Federal do Ceará, Programa de Pós Graduação em Ciências Médicas, Departamento de Medicina Clínica da Faculdade de Medicina, Fortaleza, CE, Brazil.; 8 Universidade Federal do Ceará, Faculdade de Farmácia, Odontologia e Enfermagem, Departamento de Análises Clínicas e Toxicológicas, Fortaleza, CE, Brazil.

**Keywords:** Coronary Artery Bypass, Acute Kidney Injury, Cardiopulmonary Bypass, Nephrin, Neutrophil Gelatinase-Associated Lipocalin, Lipocalin-2 Protein, NGAL Protein, Lipocalin-2

## Abstract

**Introduction:**

Cardiopulmonary bypass (CPB) for coronary artery bypass grafting (CABG) often causes kidney dysfunction and increases morbidity and mortality.

**Aims:**

To evaluate the effects of CPB on kidney structures of patients submitted to CABG using serum and urinary biomarkers.

**Methods:**

This prospective study included patients who underwent CABG over a 14-month period. Data related to clinical, surgical, and laboratory were collected. The glomerular filtration rate was estimated using the CKD-EPI equation. The urinary biomarkers trialed were nephrin, KIM-1, MCP-1, Syndecan-1, and NGAL.

**Results:**

Out of 30 patients enrolled, 22 were assessed. The mean age was 65 years and most were male. During CABG, the On-pump group had increased urinary nephrin (p = 0.007) and urinary (p = 0.036) and serum NGAL (p = 0.030) levels compared to the Off-pump group. Moreover, intraoperatively, in the On-Pump clusters, the urinary NGAL was correlated with the decrease of glomerular filtration rate in the first 48 hours after CABG (Rho = − 0.838, p = 0.009). There was no statistical difference in clinical and surgical aspects between groups according to use of CBP during CABG.

**Conclusion:**

CBP procedure used during CABG was associated with relevant effects on kidney structure, such as podocyte and tubular injury. Urinary NGAL was able to predict an impairment of glomerular filtration 48 hours after CABG.

## Introduction

Globally, cardiovascular diseases, particularly ischemic heart disease and stroke, are leading causes of death. A significant proportion of these deaths occur in low- and middle-income countries, and this trend is expected to increase in the coming years^1^. Acute kidney injury (AKI) is the most common complex postoperative complication in cardiac surgery^2^, with an incidence ranging from 7% to 40%^3^. Severe AKI is independently associated with 3- to 8-fold increase in perioperative mortality^4^, prolonged ICU and hospital stays, and increased healthcare costs^5^. Even with complete kidney function recovery, the risk of death remains high for 10 years after surgery-associated AKI^6^.

Cardiopulmonary bypass (CPB) is a widely used procedure in coronary artery bypass surgery (CABG), which is considered the gold standard for treating complex coronary artery disease^7,8^. AKI is often underdiagnosed during cardiac surgery owing to reliance on creatinine and urine output criteria, which may not accurately reflect kidney function when CPB is managed^9^. Scientific evidence suggests that avoidance of CPB using “off-pump” techniques is associated with lower rates of AKI and should therefore be considered based on individual patient risk stratification^10^.

New biomarkers have proven useful in diagnosing kidney injury, even in the absence of acute dysfunction. Urinary biomarker measurements, which detect the release of tubular and glomerular proteins in the urine during ischemic or nephrotoxic damage, may offer greater specificity and sensitivity than serum measurements. These biomarkers play a crucial role in patient prognosis and risk stratification following cardiac surgery^11,12,13,14^.

This study aimed to evaluate the impact of CPB on systemic and new urinary biomarker levels related to endothelial and kidney damage in patients undergoing CABG.

## Methods

### Study Design and Groups

This prospective study enrolled patients who underwent cardiac surgery between August 2021 and October 2022 at the Walter Cantidio University Hospital (HUWC) of Federal University of Ceara in Fortaleza, Brazil. The study was submitted to the Brazilian National research system called Plataforma Brasil under number 3.592.596, inform consent was obtained from the subjects or their legally authorized representatives, and the protocol complied with the Declaration of Helsinki.

### Selection Criteria

Patients were categorized into two groups based on the use of CPB. One cluster underwent CABG without the use of CPB bypass, a beating heart procedure (**Off-pump group**), and in the other cluster, CPB was used (**On-pump group**). The groups were divided into two groups with and without CPB to evaluate the size of the effect of the surgical intervention.

### Inclusion Criteria

1. CAD suitable for revascularization.
2. Both sexes: men and women who were not pregnant.
3. Age ≥18 years.

### Exclusion Criteria

1. Pre-existing kidney disease and exposure to nephrotoxic drugs.
2. Urine samples not available during study periods.
3. Non-CABG combined heart surgery (valvular; aorta).
4. History of more than 1 coronary bypass operation.
5. Previous heart, kidney, liver, or lung transplantation.

### Clinical, Surgical, and Laboratorial Data

1. Socio-demographic parameters: age, color, naturalness, origin, level of education, and occupation.
2. Risk assessment systems and imaging tests: Syntax score, STS score, coronary angiogram and echocardiogram.
3. Surgical parameters: myocardial protection/cardioplegia, induced hypothermia, and cardiopulmonary bypass time.

Lab tests included hemoglobin, hematocrit, electrolytes, leucocytes, platelets, blood urea, and serum creatinine; the latter two were assessed preoperative, postoperative (immediate; 24 and 48 hours), and at hospital discharge. The glomerular filtration rate (GFR) was estimated using the CKD-EPI (Chronic Kidney Disease Epidemiology Collaboration) equation^15^. Moreover, the length of the hospital stay and time from surgery to hospital discharge were registered.

### On-Pump Group: CABG Procedure and Sample Collection

Patients were placed in the supine position, underwent pre-anesthetic monitoring, and peripheral and central accesses were performed, and submitted to the collection of the first sample. The surgical procedure followed the mainstream CABG technique with the installation of CPB^7^ before the marking of coronary artery bypass graft.

Distal and proximal anastomosis were performed, and at this time the second blood sample for biomarker analysis was collected. After completing the revascularization, the patient was transferred to the intensive care unit, and finally the third sample was collected for biomarker analysis.

### Off-Pump Group: CABG Procedure and Sample Collection

For this group, the main difference from the other cluster was that the procedure was performed with a beating heart directly without CPB. The first, second, and third sample collections were carried out in the same way as the other group. However, the second sample was collected after graft revascularization without the installation of CPB.

### Measurements of Kidney and Endothelial Biomarkers

Blood samples were collected in tubes containing separating gel and sent to the support laboratory for centrifugation to obtain the serum used for measuring the biomarkers as recommended in the leaflet of the ELISA R&D System kit. Blood and urine samples were collected from all patients at three distinct study periods:

1. Preoperative period;
2. Intraoperative period – right after coronary anastomoses;
3. Postoperative period – at ICU admission.

Samples were conditioned at 4ºC and then transferred within 2 h to an ultrafreezer at −80ºC. All biomarkers were quantified in urine and blood samples using enzyme-linked immunosorbent assay (ELISA). Nephrin, MCP-1 (Monocyte chemoattractant protein-1), KIM-1 (Kidney injury molecule-1), and NGAL (Neutrophil gelatinase-associated lipocalin) were quantified in the urine sample. NGAL and syndecan-1 (endothelial glycocalyx damage biomarker) were evaluated in serum. The levels of urinary biomarkers were adjusted for urinary creatinine.

**Figure 1:**
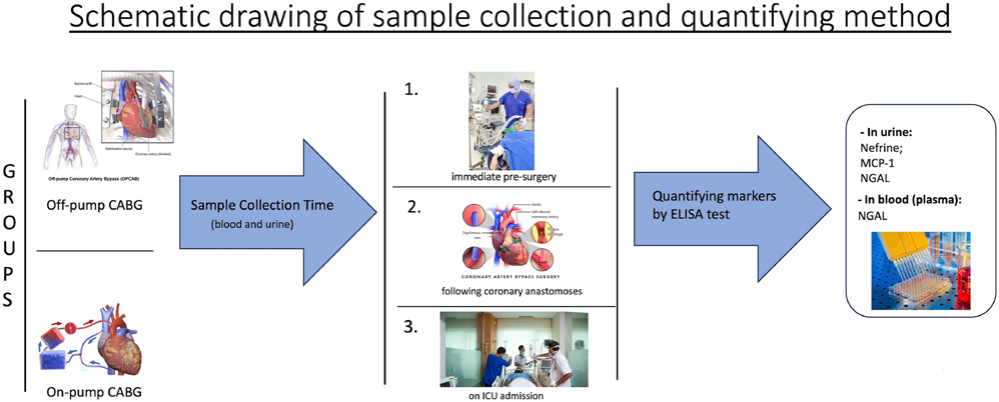
Measurements of kidney and endothelial biomarkers. Notes: Images obtained from public sources and under Creative Commons license: [1] https://images.app.goo.gl/hVp4Kq5j3sosjRiX7; [2] https://images.app.goo.gl/z2RinjcSy1JAVZFb9; [3] https://images.app.goo.gl/U5FMjAxiBUx8S2ok6; [4] https://commons.wikimedia.org/w/index.php?curid=12484442; [5] https://commons.wikimedia.org/wiki/File:Blausen_0468_Heart-Lung_Machine.png; [6] https://commons.wikimedia.org/w/index.php?curid=4489929.

Specific ELISA kits were used for the following measurements: Nephrin (R&D Systems^®^: cat# DY4269), MCP-1 (R&D Systems^®^: cat# DY479), KIM-1 (R&D Systems^®^: cat# DY1750), NGAL (R&D Systems^®^: cat# DY1757), and Syndecan-1 (Abcam^®^ ab47352). The procedures followed the manufacturer’s recommendations and are portrayed in the graphical diagram (Figure 1).

### Statistical Analysis

Categorical variables were expressed as absolute counts and percentages. Quantitative variables were tested for normal distribution using the Shapiro-Wilk test. Skewness and kurtosis of the data were analyzed using histograms and Q-Q plots. Normal data were expressed as mean ± standard deviation and non-normal data as median and interquartile range. For comparisons between categorical data, Chi-square and Fisher’s exact tests were used, as appropriate. In the paired analysis with comparisons between dependent groups according to the surgery periods, the Friedman test was used for non-normal data. In case of statistical significance, the Wilcoxon test was used for pairwise comparisons, where the critical value of statistical significance was divided by the number of matched groups (three periods): to account for multiple comparisons and reduce the risk of type I error, a p-value threshold of 0.016 was applied for statistical significance. For the other tests, p < 0.05 was considered statistically significant. Data were analyzed using the SPSS software for Macintosh, version 23 (Armonk, NY: IBM Corp).

## Results

A total of 30 patients were enrolled, but 8 patients were excluded due to a variety of reasons: lost to follow-up, late diagnosis of chronic renal failure, and refusal to continue recording information for research. Therefore, 22 patients remained in the study. Their mean age was 65 ± 8 years and 68% were men. Arterial hypertension (91%) and diabetes (36%) were highly prevalent comorbidities. Smoking was present in 23%, and familial history of CAD was seen in an equal percentage of the patients. The most frequent Syntax score was 3 (91%) (Table 1).

**Table 1:** Clinical and surgical aspects and preoperative hospitalization of the included patients

	Total group (n = 22)
Clinical aspects	
Age, years	65 ± 8
Male, gender	15 (68.2)
Ethnicity	
White	3 (13.6)
Brown	19 (86.4)
Comorbidities and risk factors	
Arterial hypertension	20 (90.9)
Diabetes	8 (36.4)
Smoking	5 (22.7)
Familiar history coronopathy	5 (22.7)
Obesity	1 (4.5)
Dyslipidemia	11 (50)
Others	7 (31.8)
Syntax score	
1	1 (4.5)
2	1 (4.5)
3	19 (91)
STS score	0.92 (0.58–2.58)
FEVEs	52 ± 8
Surgical aspects and hospitalization	
CPB	
No	13 (59.1)
Yes	9 (40.9)
CPB time (min)	86 ± 27
Hypothermia	6 (27.7)
Length of hospital stay, days	9 (5–12)
Time from surgery to hospital discharge, days	7 (5–10)

Abbreviations – CPB: cardiopulmonary bypass. Notes – Quantitative data expressed as mean ± standard deviation or median and interquartile range in parentheses. Categorical data expressed as absolute counts and percentages in parentheses.

Regarding surgical aspects, nine patients (41%) were subjected to the CPB procedure. The mean CPB duration was 86 ± 27 min. The median with interquartile range (IQR) for the length of the hospital stay was 9 (5–12) days, and the time from surgery to hospital discharge was 7 (5–10) days (Table 1). There was no statistical difference in any of the clinical aspects in independent comparisons between groups. The STS score was higher (2.52 [IQR:0.92–3.03] vs. 0.58 [IQR: 0.31–0.81], p = 0.030) and LVEF was lower (47 ± 7 vs. 59 ± 2, p = 0.013) in the On-pump group. The on-pump group had higher frequency of induced hypothermia (75% vs. 0%, p = 0.001), because lowering body temperature is part of the revascularization technique. There were no statistical differences in time from surgery to hospital discharge and in lab data collected before the surgery (Table 2).

**Table 2:** Clinical, surgical aspects and hospitalization of the included patients according to the coronary artery bypass use during surgery

	No CPB (n = 13)	CPB (n = 9)	p*
Clinical aspects			
Age, years	63 ± 7	68 ± 9	0.176
Male, gender	9 (69.2)	6 (66.7)	1.000
Ethnicity			1.000
White	2 (15.4)	1 (11.1)	
Brown	11 (84.6)	8 (88.9)	
Comorbidities and risk factors			
Arterial hypertension	12 (92.3)	8 (88.9)	1.000
Diabetes	7 (53.8)	1 (11.1)	0.074
Smoking	4 (30.8)	1 (11.1)	0.360
DAOP	0 (0)	0 (0)	–
Arrhythmias	0 (0)	0 (0)	–
Familiar history coronopathy	2 (15.4)	3 (33.3)	0.609
Obesity	0 (0)	1 (11.1)	0.409
Dyslipidemia	6 (46.2)	5 (55.6)	1.000
Others	5 (38.5)	2 (22.2)	0.648
Syntax score			0.114
1	0 (0)	1 (11.1)	
2	0 (0)	1 (11.1)	
3	12 (100)	7 (77.8)	
STS score	0.58 (0.31–0.81)	2.52 (0.92–3.03)	0.030
FEVEs	59 ± 2	47 ± 7	0.013
Hypothermia and hospital stay			
Hypothermia	0 (0)	6 (75)	0.001
Length of hospital stay, days	8 (7–12)	10 (5–11)	0.896
Time from surgery to hospital discharge, days	7 (6–11)	7 (5–8)	0.744
Laboratorial (before surgery)			
Hemoglobin (g/dL)	13.2 ± 1.9	12.7 ± 2	0.560
Hematocrit (%)	40 ± 5	38 ± 6	0.346
Sodium (mEq/L)	139 ± 3	142 ± 8	0.291
Potassium (mEq/L)	4.61 ± 0.41	4.38 ± 0.29	0.180
Leukocytes	7596 (6200–8760)	8240 (7673–12800)	0.169
Platelets	248833 ± 38442	211711 ± 64056	0.114
Blood urea (mg/dL)	42 ± 10	43 ± 18	0.780
Creatinine (mg/dL)	0.84 (0.75–1.05)	1 (0.8–1.1)	0.651
eGFR (mL/min.1.73m^2^)	89 ± 21	76 ± 30	0.266

Abbreviations – CPB: cardiopulmonary bypass. eGFR: Estimated glomerular filtration rate. Notes – Quantitative data expressed as mean ± standard deviation or as median and interquartile range in parentheses. Categorical data expressed as absolute counts and percentages in parentheses.

*For categorical data, the Fisher or chi-square test was used. For quantitative data, Student's t test was used for normal data and the Mann-Whitney test for non-normal data.

**Figure 2:**
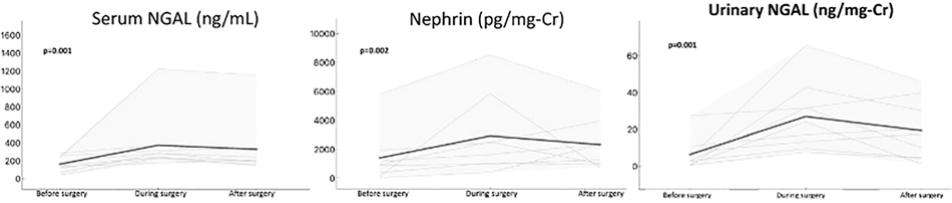
Urinary NGAL, urinary nephrin, and serum NGAL significantly increased during the surgery in On-pump group.

Regarding new biomarkers, during CAGB surgery, the CPB group showed increased levels of urinary nephrin compared to the off-pump group (2,051.42 [1,018.82–4,166.25] vs. 400 [27.59–1,186.9] pg/mg-Cr, p = 0.007). In addition, urinary and serum NGAL were higher in the pump group compared to the non-pump group (Table 2). The same effect was observed for NGAL in urine (24 [13.28–31.58] vs. 7.24 [4.55–14.18] ng/mg-Cr, p = 0.036) and serum (282.12 [232.5–312.25] vs. 165.15 [126.16–186.42] ng/mL, p = 0.030). There were no statistical differences regarding other surgical parameters (Table 3). Furthermore, in the paired comparisons, urinary nephrin, urinary NGAL, and serum NGAL were significantly increased during the surgery only in the On-pump group. These levels remained high even after the surgery, i.e., on ICU admission, compared to the “before surgery” level (Table 4 and Figure 2).

**Table 3:** Comparison of biomarker levels in each surgery period in patients who underwent or not cardiopulmonary bypass (CPB) during surgery

	No CPB (n = 13)	CPB (n = 9)	p*
Before surgery			
uNephrin (pg/mg-Cr)	341.73 (33.33–1654.98)	887.56 (381.4–1168.28)	0.556
uMCP-1 (pg/mg-Cr)	35 (10.09–56.88)	13.62 (1.7–129.7)	0.794
uKIM-1 (pg/mg-Cr)	3860 (842–8245)	9651 (464–66589)	0.473
uNGAL (ng/mg-Cr)	5.17 (2.5–6.51)	2.86 (0.84–7.76)	0.431
sNGAL (ng/mL)	90.72 (74.77–136.79)	142.11 (117.3–234.27)	0.393
Syndecan-1 (ng/mL)	75.21 (68.5–99.49)	75.85 (68.18–86.39)	0.601
During surgery			
uNephrin (pg/mg-Cr)	400 (27.59–1186.9)	2051.42 (1018.82–4166.25)	**0.007**
uMCP-1 (pg/mg-Cr)	16.08 (2.33–154.65)	4.25 (2.01–47.65)	0.492
uKIM-1 (pg/mg-Cr)	2616 (966–13506)	7330 (2978–28377)	0.460
uNGAL (ng/mg-Cr)	7.24 (4.55–14.18)	24 (13.28–31.58)	**0.036**
sNGAL (ng/mL)	165.15 (126.16–186.42)	282.12 (232.5–312.25)	**0.030**
Syndecan-1 (ng/mL)	126.34 (93.42–367.92)	116.11 (77.44–287.08)	0.393
After surgery (ICU admission)			
uNephrin (pg/mg-Cr)	1054.84 (190.05–3384.75)	1806.52 (958.33–3142.08)	0.395
uMCP-1 (pg/mg-Cr)	2.39 (1.21–35.75)	2.03 (0.94–46.58)	0.762
uKIM-1 (pg/mg-Cr)	4242 (20–41253)	1584 (254–21520)	0.840
uNGAL (ng/mg-Cr)	7.51 (5.37–18.18)	17.38 (4.57–30.15)	0.431
sNGAL (ng/mL)	189.96 (129.71–253.76)	207.68 (186.42–324.65)	0.431
Syndecan-1 (ng/mL)	88.31 (66.58–227.96)	80.96 (73.93–161.49)	0.984

Abbreviations – CPB: cardiopulmonary bypass. Notes – Quantitative data expressed as median and interquartile range in parentheses.

*The Mann-Whitney test was used.

**Table 4:** Paired comparison of biomarker levels between each study period in patients who underwent cardiopulmonary bypass or not

	Periods			p*
	Before Surgery	During Surgery	After surgery (ICU admission)	
CPB group				
uNephrin (pg/mg-Cr)	887.56 (381.4–1168.28)	2051.42 (1018.82–4166.25)	1806.52 (958.33–3142.08)	**0.002A**
uMCP-1 (pg/mg-Cr)	13.62 (1.7–129.7)	4.25 (2.01–47.65)	2.03 (0.94–46.58)	0.135
uKIM-1 (pg/mg-Cr)	9651 (464–66589)	7330 (2978–28377)	1584 (254–21520)	0.867
uNGAL (ng/mg-Cr)	2.86 (0.84–7.76)	24 (13.28–31.58)	17.38 (4.57–30.15)	**0.001A**
sNGAL (ng/mL)	142.11 (117.3–234.27)	282.12 (232.5–312.25)	207.68 (186.42–324.65)	**0.001B**
Syndecan-1 (ng/mL)	75.85 (68.18–86.39)	116.11 (77.44–287.08)	80.96 (73.93–161.49)	0.717
Patients without CPB				
uNefrin (pg/mg-Cr)	341.73 (33.33–1654.98)	400 (27.59–1186.9)	1054.84 (190.05–3384.75)	0.092
uMCP-1 (pg/mg-Cr)	35 (10.09–56.88)	16.08 (2.33–154.65)	2.39 (1.21–35.75)	0.459
uKIM-1 (pg/mg-Cr)	3860 (842–8245)	2616 (966–13506)	4242 (20–41253)	0.717
uNGAL (ng/mg-Cr)	5.17 (2.5–6.51)	7.24 (4.55–14.18)	7.51 (5.37–18.18)	0.500
sNGAL (ng/mL)	90.72 (74.77–136.79)	165.15 (126.16–186.42)	189.96 (129.71–253.76)	0.063
Syndecan-1 (ng/mL)	75.21 (68.5–99.49)	126.34 (93.42–367.92)	88.31 (66.58–227.96)	0.092

Abbreviations – CPB: cardiopulmonary bypass. Notes – Quantitative data expressed as median and interquartile range in parentheses.

*Friedman test was used. A: Significance observed between “Before surgery” vs. other periods. B: Significance observed between all periods.

**Figure 3:**
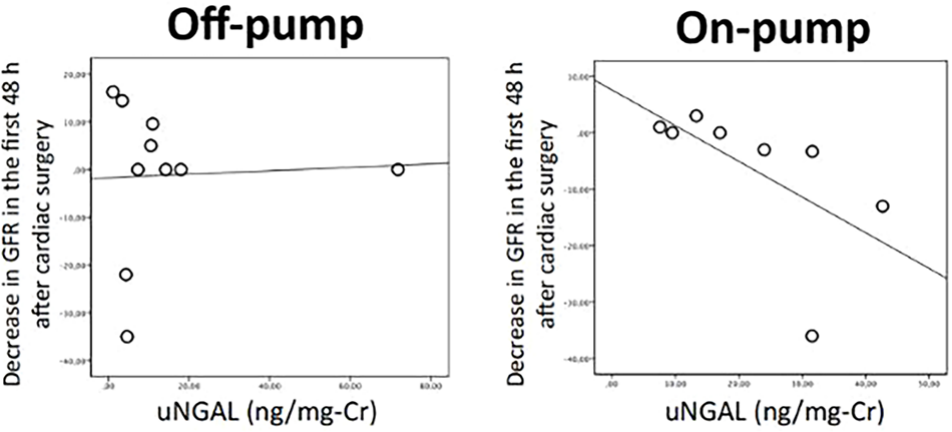
Correlation between uNGAL level during CABG and variation in GFR in the first 48h post-surgery.

Finally, increasing urinary NGAL levels during surgery in patients undergoing CPB (On-pump) was correlated with decreasing GFR in the first 48 hours after cardiac surgery (Rho = −0.838, p = 0.009). However, no significant correlation was observed in the Off-pump group (Figure 3).

## Discussion

In the present study, we have shown that CPB used during coronary artery bypass grafting was associated with damage to glomerular and tubular structures of the kidney and further reduction in GFR 48 hours postoperatively. Urinary biomarkers were useful to show that CPB use on On-pump CABG was associated with further kidney disease development in comparison to Off-pump surgery, and these patients should be monitored on postoperative period to prevent kidney complications.

The pathogenesis of postoperative AKI is multifactorial and involves diverse mechanisms, such as renal hypoperfusion, ischemia and reperfusion injury, neurohumoral activation, inflammation, oxidative stress, nephrotoxins, and mechanical factors^10,16^. Moreover, patients with previous kidney dysfunction have higher risk of developing AKI after surgery, which is associated with poor prognosis and death^9^. The early and precise diagnosis of kidney dysfunction may improve the risk stratification of AKI after CABG surgery, and allow appropriate prevention and treatment strategies^3^. In a systematic review of 33 randomized controlled studies with more than 1700 patients undergoing CAGB, subjects undergoing off-pump procedures were associated with a reduced risk of AKI. Despite that, the ROOBY study with 2203 patients did not find any survival benefit or renoprotective effect in patients undergoing off-pump revascularization compared with patients who used circulatory assistance with bypass, including a higher rate of cardiac death and lower graft patency in the first year of follow-up. We speculate that CPB procedure may potentially cause an important damage on kidney structure, leading for acute GFR reduction^17^. In a recent study with HFrEF patients in decompensated acute state that underwent on-pump CABG, Simão et al.^18^ showed an inflammatory response, and suggested that the exacerbation of the pro-inflammatory state secondary to acute disease and CPB had a devastating synergistic effect, which contributed to On-pump patients death.

In this present study, the patients submitted to on-pump CABG presented increased levels of urinary and serum NGAL compared to off-pump CABG, mostly in the intraoperative period. Moreover, levels of urinary NGAL during the intraoperative period of CAGB surgery with the support of CPB were associated with further GFR reduction. NGAL is typically a secretory protein of low molecular weight originally identified in neutrophils and later found to be present in other organs, including kidneys^19,20^. Studies have identified NGAL as one of the most surprisingly regulated genes (and proteins overexpressed in the kidney after ischemia)^21^.

The first studies with NGAL as an early AKI biomarker in biological samples were performed in the context of cardiac surgery^22^, and then several studies were performed to investigate the diagnostic performance of urinary and blood NGAL for AKI. A surgery-associated NGAL cardiac score was even proposed and also used in a decision algorithm for surgery modifications, including rescheduling the surgery^11^.

In a meta-analysis with 16 studies evaluating the utility of NGAL for AKI diagnosis following cardiac surgery requiring CPB, the diagnostic value of plasma NGAL was inconclusive and needed more investigation^23^. The diagnostic performance of NGAL assessed 4–8 h following cessation of CPB in cardiac surgery patients was superior to NGAL assessed at less than 4 h and 24 h for the early AKI. Despite the potential role for the diagnostic utility of NGAL in this clinical setting, there are few studies, which have substantial heterogeneity and weak conclusions^23^. Hence, the present study is relevant in distinguishing the NGAL involvement in a specific cardiac surgery (Coronary revascularization - CABG) with CPB compared to the off-pump surgery, proving that alterations in NGAL levels, either in urine or blood, were associated with CPB surgery.

This study showed for the first time that levels of urinary nephrin were elevated during the intraoperative period of cardiac surgery, especially in the On-pump group. Nephrin is the first transmembrane globulin protein located in the filtration slit diaphragm, which plays a key role on podocyte foot processes related to selective permeability of the glomerular filtration barrier, avoiding protein linkage^24^. Consequently, in a pioneering way, it demonstrates that this protein works as an early marker of podocyte injury in CBP-mediated AKI. Experimental studies have shown that alterations in nephrin expression were associated with hypertension and antihypertensive treatment response^25^. In cells, downregulated expression of nephrin has been reported to indicate a lesion in podocytes, which causes the disruption of foot processes and of the slit diaphragm^26^. As a consequence, nephrinuria was associated with detachment of podocytes leading to podocytopenia and then glomerulosclerosis^27^. In another study with diabetic patients divided according to albuminuria levels, the urinary nephrin levels were correlated negatively with GFR, indicating that nephrinuria is a marker of disordered renal function^28^. In the same study, it was suggested that nephrinuria may be more useful as early biomarker of CKD than albuminuria.

To our knowledge, there are no studies evaluating urinary nephrin levels in the context of human cardiac surgery. In an experimental model of ischemia-reperfusion acute kidney injury, podocyte injury was an important target and the 30-min ischemia-reperfusion injury was associated with decreased nephrin expression in podocytes, increased proteinuria, renal function decline, and increased fibrosis markers, which contribute to permanent renal cells dysfunction^29^.

In the present research, the elevated levels of nephrin may reflect podocyte injury not only in cardiac surgery but specifically in CABG without the on-pump procedure, and these levels should be monitored to investigate further kidney complications related with fibrosis mechanisms, as in CKD.

The urinary MCP-1 levels evaluated in this present study on ICU admission after cardiac surgery were significantly correlated with decreased GFR in the first 48 hours in the CPB group. Urinary MCP-1 is a well-recognized biomarker of CKD and has been associated with fibrosis mechanisms^30,31^. In addition, MCP-1 appears to contribute to kidney inflammation and nephrin expression alterations, leading to podocyte injury and clinical manifestations such as proteinuria, albuminuria, and decreased GFR^32,33^. Thus, we speculated that ischemia-reperfusion injury induced by on-pump CABG could activate podocyte injury and inflammatory mechanisms that persist after surgery and may contribute to kidney injury and decreased GFR in the first 48 hours.

On the international context, CKD prevalence and incidence rates are increasing, mainly among adults over 30 years of age, according to the Brazilian Society of Nephrology. Given that the treatment of CKD is expensive and burdens health systems, an early and correct diagnosis of AKI is of social importance for the adoption of therapeutic measures in a timely manner or correction of potentially reversible causes, providing opportunities for prevention or limiting the progression of kidney damage to a CKD that requires specialized support. In this sense, non-conventional biomarkers such as NGAL, MCP-1, KIM-1, and nephrin are described in recent studies as promising for the early assessment of AKI due to their high sensitivity, showing a significant increase in several clinical contexts even before the manifestation of classic signs and symptoms of AKI^34^.

Previous research has highlighted the importance of using non-conventional biomarkers for the early diagnosis of AKI in premature newborns diagnosed with sepsis, helping to make more assertive decisions and avoid later therapeutic management of these patients^35^. Furthermore, as clinical situations such as COVID-19, which is a pro-inflammatory and systemic infection, AKI manifests as a complication that worsens the patient’s clinical condition, which can lead to loss of function and death, and the use of biomarkers, especially urinary NGAL, have proven useful for predicting the prognosis of the infected patients^36^. Renal function was correlated with hepatosplenic schistosomiasis (HSS), indicating that renal dysfunction is related to HSS, mostly tubular dysfunction, verified by urinary concentration and incomplete distal acidification, with urine MCP-1 being a more sensitive biomarker than the urinary albumin excretion rate^37^.

This study had limitations. It was single-centered and had a small sample size, which hindered regression analysis. Moreover, other urinary biomarkers such as proteinuria and albuminuria were not evaluated, which could help to evaluate glomerular structure alterations. The use of CPB during cardiac surgery was associated with structural kidney damage, including podocyte and tubular injury, as well as short-term kidney injury. Moreover, the statistical analysis was primarily univariate, preventing us from conclusively attributing the increase in biomarker levels solely to CPB in a small sample of nine patients. Other perioperative factors may have influenced the observed associations. Additionally, determining the required sample size to confirm the magnitude of the effect of CPB would require a formal statistical power analysis. Future studies with larger cohorts and multivariate analysis are needed to better isolate the independent impact of CPB on biomarker changes.

In conclusion, novel biomarkers may be useful in the early detection of CKD, in which urinary NGAL was able to anticipate a 48-hour CABG-mediated impairment of glomerular filtration.

## Data Availability

The data used in this study are not publicly available due to confidentiality agreements, as stipulated by the ethics committee, the participants’ consent, or the responsible institution. Access to the data may be considered upon formal request and prior approval from the relevant parties, in accordance with confidentiality requirements and the protection of sensitive information.

## Supplementary Material

The following online material is available for this article:

Table S1 – Correlation analysis between biomarkers and glomerular filtration rate in the first 48 hours after surgery.
