# Supplementary material for: Urinary and serum biomarkers of renal injury in coronary artery bypass grafting: a prospective evaluation with new biomarkers’
Source: J Bras Nefrol. 2025 Jul 18;47(3):e20240173. doi: 10.1590/2175-8239-JBN-2024-0173en (PMC12279285; doi:10.1590/2175-8239-JBN-2024-0173en)
Supplement: Supplementary file 1 [file 2175-8239-jbn-47-3-e20240173-suppl1.pdf]

Braz. J. Nephrol.
DOI: https://doi.org/10.1590/2175-8239-JBN-2024-0173en

Supplementary Material to "Urinary and serum biomarkers of renal injury in coronary artery bypass grafting: a prospective evaluation with new biomarkers"

Table S1. Correlation analysis between biomarkers and glomerular filtration rate variation in the first 48 hours after surgery.

	Total group (n=22)		CPB (n=9)		Without CPB (n=13)	
	eGFR variation in first 48 hours		eGFR variation in first 48 hours		eGFR variation in first 48 hours	
	rho	*p*	*rho*	*p*	*rho*	*p*
**Before surgery**						
uNefrina (pg/mg-Cr)	0.081	0.75	0.383	0.349	0.013	0.973
uMCP-1 (pg/mg-Cr)	-0.384	0.116	-0.766	**0.027**	-0.213	0.555
uKIM-1 (pg/mg-Cr)	0.285	0.284	0.257	0.089	0.221	0.567
uNGAL (ng/mg-Cr)	-0.014	0.957	0.192	0.649	-0.419	0.228
sNGAL (ng/mL)	0.156	0.28	0.24	0.568	0.138	0.4
Syndecan-1 (ng/mL)	0.39	0.11	0.18	0.67	0.463	0.178
**During surgery**						
uNefrina (pg/mg-Cr)	-0.099	0.717	-0.342	0.452	0.305	0.425
uMCP-1 (pg/mg-Cr)	-0.143	0.596	-0.541	0.21	0,000	1,000
uKIM-1 (pg/mg-Cr)	0.134	0.633	0.631	0.129	0.036	0.933
uNGAL (ng/mg-Cr)	-0.419	0.083	-0.838	**0.009**	-0.281	0.431
sNGAL (ng/mL)	0.091	0.718	-0.299	0.471	0.425	0.221
Syndecan-1 (ng/mL)	0.246	0.438	0.599	0.117	0.137	0.21
**ICU admission after surgery**						
uNefrina (pg/mg-Cr)	0.133	0.624	0.198	0.67	0.409	0.274
uMCP-1 (pg/mg-Cr)	-0.515	**0.05**	-0.793	**0.033**	-0.533	0.174
uKIM-1 (pg/mg-Cr)	0.139	0.608	0.396	0.379	0.102	0.794
uNGAL (ng/mg-Cr)	-0.343	0.164	-0.299	0.471	-0.394	0.26
sNGAL (ng/mL)	0.206	0.412	0.132	0.756	0.413	0.236
Syndecan-1 (ng/mL)	-0.39	0.11	-0.275	0.509	-0.488	0.153

Abbreviations - eGFR: estimated glomerular filtration rate. CPB: cardiopulmonary bypass.
