# Supplementary material for: Urinary and serum biomarkers of renal injury in coronary artery bypass grafting: a prospective evaluation with new biomarkers’
Source: J Bras Nefrol. 2025 Jul 18;47(3):e20240173. doi: 10.1590/2175-8239-JBN-2024-0173en (PMC12279285; doi:10.1590/2175-8239-JBN-2024-0173en)
Supplement: Translation

# Translation: Biomarcadores urinários e séricos de injúria renal na cirurgia de revascularização do miocárdio: uma avaliação prospectiva com novos biomarcadores

**DOI:** 10.1590/2175-8239-JBN-2024-0173pt

**Authors:** Antônio Felipe Leite Simão, Gdayllon Cavalcante Meneses, Lia Cavalcante Cezar, Letícia Machado de Araújo, Alice Maria Costa Martins, Heraldo Guedis Lobo, Bruna Viana Barroso Martins, Geraldo Bezerra da Silva, Elizabeth De Francesco Daher, José Glauco Lobo

**Affiliations:** 1 Universidade Federal do Ceará, Hospital Universitário Walter Cantidio, Fortaleza, CE, Brasil.; 2 Universidade Federal do Ceará, Faculdade de Medicina, Departamento de Fisiologia e Farmacologia, Programa de Pós-Graduação em Ciências Médicas, Fortaleza, CE, Brasil.; 3 Universidade Federal do Ceará, Faculdade de Medicina, Departamento de Cirurgia, Programa de Pós-Graduação em Ciências Médicas, Fortaleza, CE, Brasil.; 4 Hospital de Messejana Dr. Carlos Alberto Studart Gomes, Fortaleza, CE, Brasil.; 5 Xiamen University, Xiamen Cardiovascular Hospital, Xiamen, China.; 6 Universidade Federal do Ceará, Faculdade de Medicina, Departamento de Imunologia, Fortaleza, CE, Brasil.; 7 Universidade Federal do Ceará, Programa de Pós Graduação em Ciências Médicas, Departamento de Medicina Clínica da Faculdade de Medicina, Fortaleza, CE, Brasil.; 8 Universidade Federal do Ceará, Faculdade de Farmácia, Odontologia e Enfermagem, Departamento de Análises Clínicas e Toxicológicas, Fortaleza, CE, Brasil.

**Keywords:** Cirurgia de Revascularização do Miocárdio, Injúrias Renais Agudas, Circulação Extracorpórea, Nefrina, Lipocalina Associada a Gelatinase de Neutrófilo, Proteína Lipocalina-2, Proteína NGAL, Lipocalina-2

## Abstract

**Introdução:**

A circulação extracorpórea (CEC) para cirurgia de revascularização do miocárdio (CRM) frequentemente causa disfunção renal e aumento da morbimortalidade.

**Objetivos:**

Avaliar os efeitos da CEC nas estruturas renais de pacientes submetidos à CRM, por meio de biomarcadores séricos e urinários.

**Métodos:**

Este estudo prospectivo incluiu pacientes submetidos à CRM em um período de 14 meses. Foram coletados dados clínicos, cirúrgicos e laboratoriais. A taxa de filtração glomerular foi estimada pela equação CKD-EPI. Os biomarcadores urinários avaliados foram nefrina, KIM-1, MCP-1, Syndecan-1 e NGAL.

**Resultados:**

Dos 30 pacientes inscritos, 22 foram avaliados. A idade média foi de 65 anos e a maioria era do sexo masculino. Durante a CRM, o grupo *On-pump* apresentou níveis aumentados de nefrina urinária (p = 0,007), NGAL urinária (p = 0,036) e sérica (p = 0,030) em comparação ao grupo *Off-pump*. Além disso, no período intraoperatório, nos grupos *On-pump*, a NGAL urinária foi correlacionada com a redução da taxa de filtração glomerular nas primeiras 48 horas após a CRM (Rho = −0,838; p = 0,009). Não houve diferença estatística entre os grupos quanto aos aspectos clínicos e cirúrgicos de acordo com o uso da CEC durante a CRM.

**Conclusão:**

O uso do procedimento de CEC durante a CRM esteve associado a efeitos relevantes na estrutura renal, como lesão tubular e de podócitos. A NGAL urinária foi capaz de predizer o comprometimento da filtração glomerular 48 horas após a CRM.

## Introdução

Globalmente, as doenças cardiovasculares, em especial a cardiopatia isquêmica e o acidente vascular cerebral, constituem as principais causas de óbito. Uma proporção significativa desses óbitos ocorre em países de baixa e média renda, e espera-se que essa tendência aumente nos próximos anos^1^. A injúria renal aguda (IRA) é a complicação pós-operatória mais comum em cirurgias cardíacas^2^, com uma incidência que varia de 7% a 40%^3^. A IRA grave está independentemente associada a um aumento de 3 a 8 vezes na mortalidade perioperatória^4^, ao prolongamento do tempo de permanência na UTI e no hospital e ao aumento dos custos de saúde^5^. Mesmo com a recuperação completa da função renal, o risco de óbito permanece elevado por 10 anos após um quadro de IRA associada à cirurgia^6^.

A circulação extracorpórea (CEC) é um procedimento amplamente utilizado na cirurgia de revascularização do miocárdio (CRM), considerada o padrão-ouro para o tratamento da doença arterial coronariana complexa^7,8^. A IRA é frequentemente subdiagnosticada durante a cirurgia cardíaca devido à dependência dos critérios de creatinina e débito urinário, que podem não refletir com precisão a função renal quando a CEC é realizada^9^. Evidências científicas sugerem que a não utilização de CEC, por meio de técnicas “off-pump”, está associada a taxas mais baixas de IRA e, portanto, deve ser considerada com base na estratificação de risco individual do paciente^10^.

Novos biomarcadores têm se mostrado úteis no diagnóstico de injúria renal, mesmo na ausência de disfunção aguda. As medições de biomarcadores urinários, que detectam a liberação de proteínas tubulares e glomerulares na urina durante lesões isquêmicas ou nefrotóxicas, podem oferecer maior especificidade e sensibilidade do que as medições séricas. Esses biomarcadores desempenham um papel crucial no prognóstico do paciente e na estratificação de risco após a cirurgia cardíaca^11–14^.

O objetivo deste estudo foi avaliar o impacto da CEC nos níveis sistêmicos, bem como de novos biomarcadores urinários relacionados a danos endoteliais e renais em pacientes submetidos a CRM.

## Métodos

### Desenho do Estudo e Grupos

Este estudo prospectivo incluiu pacientes sub­metidos à cirurgia cardíaca entre agosto de 2021 e outubro de 2022 no Hospital Universitário Walter Cantídio (HUWC) da Universidade Federal do Ceará em Fortaleza, Brasil. O estudo foi submetido ao sistema nacional de registro de pesquisas brasileiro denominado Plataforma Brasil, sob o número 3.592.596. O consentimento informado foi obtido dos participantes ou de seus representantes legais autorizados, e o protocolo estava em conformidade com a Declaração de Helsinque.

### Critérios de Seleção

Os pacientes foram categorizados em dois grupos com base no uso da CEC. Um grupo foi submetido à CRM sem o uso de CEC – procedimento com o coração batendo (**grupo** ***on-pump***); enquanto no outro grupo a CEC foi empregada (**grupo** ***off-pump***). A divisão dos grupos em com CEC e sem CEC foi feita para avaliar a magnitude do efeito da intervenção cirúrgica.

### Critérios de Inclusão

1. DAC indicada para revascularização.
2. Ambos os sexos: homens e mulheres não gestantes.
3. Idade ≥18 anos.

### Critérios de Exclusão

1. Doença renal preexistente e exposição a medicamentos nefrotóxicos.
2. Amostras de urina não disponíveis durante os períodos do estudo.
3. Cirurgia cardíaca combinada não-CRM (valvar; aorta).
4. Histórico de mais de uma cirurgia de revascularização do miocárdio.
5. Transplante prévio de coração, rim, fígado ou pulmão.

### Dados Clínicos, Cirúrgicos e Laboratoriais

1. Parâmetros sociodemográficos: idade, cor, naturalidade, origem, nível de escolaridade e ocupação.
2. Sistemas de avaliação de risco e exames de imagem: Escore Syntax, STS, cinecoro­nariografia e ecocardiograma.
3. Parâmetros cirúrgicos: proteção miocárdica/cardioplegia, hipotermia induzida e tempo de circulação extracorpórea.

Os exames laboratoriais incluíram hemoglobina, hematócrito, eletrólitos, leucócitos, plaquetas, ureia sanguínea e creatinina sérica; os dois últimos foram avaliados no pré-operatório, no pós-operatório (imediato; 24 e 48 horas) e na alta hospitalar. A taxa de filtração glomerular (TFG) foi estimada por meio da equação CKD-EPI (*Chronic Kidney Disease Epidemiology Collaboration*)^15^. Além disso, foram registrados o tempo de permanência hospitalar e o intervalo entre a cirurgia e a alta hospitalar.

### Grupo *on-Pump*: Procedimento de CRM e Coleta de Amostras

Os pacientes foram posicionados em decúbito dorsal, submetidos à monitorização pré-anestésica e foram realizados os acessos periférico e central. O procedimento cirúrgico seguiu a técnica convencional de CRM com a instalação de CEC^7^ antes da marcação do enxerto de *bypass* da artéria coronária.

Foram realizadas as anastomoses distal e proximal, momento em que foi coletada a segunda amostra de sangue para análise de biomarcadores. Após a conclusão da revascularização, o paciente foi transferido para a unidade de terapia intensiva, onde finalmente foi coletada a terceira amostra para análise de biomarcadores.

### Grupo *Off-Pump*: Procedimento de CRM e Coleta de Amostras

Para este grupo, a principal diferença em relação ao outro grupo foi que o procedimento foi realizado com o coração batendo, diretamente, sem CEC. As coletas da primeira, segunda e terceira amostras foram realizadas da mesma forma que no outro grupo. No entanto, a segunda amostra foi coletada após a revascularização do enxerto, sem a instalação da CEC.

### Medições de Biomarcadores Renais e Endoteliais

As amostras de sangue foram coletadas em tubos contendo gel separador e encaminhadas ao laboratório de apoio para centrifugação, a fim de obter o soro utilizado para medição dos biomarcadores, conforme as recomendações contidas na bula do kit ELISA R&D System. Foram coletadas amostras de sangue e urina de todos os pacientes em três períodos distintos do estudo:

1. Período pré-operatório;
2. Período intraoperatório – logo após as anastomoses coronárias;
3. Período pós-operatório - na admissão na UTI.

As amostras foram acondicionadas a 4ºC e, em seguida, transferidas em um prazo de 2 horas para um ultrafreezer a −80ºC. Todos os biomarcadores foram quantificados em amostras de urina e sangue por meio de ensaio de imunoabsorção enzimática (ELISA). Nefrina, MCP-1 (proteína quimioatraente de monócitos-1), KIM-1 (molécula de lesão renal-1) e NGAL (lipocalina associada à gelatinase de neutrófilos) foram quantificadas na amostra de urina. NGAL e Syndecan-1 (biomarcador de dano ao glicocálix endotelial) foram avaliados no soro.

Foram utilizados kits ELISA específicos para as seguintes medições: Nefrina (R&D Systems^®^: cat# DY4269), MCP-1 (R&D Systems^®^: cat# DY479), KIM-1 (R&D Systems^®^: cat# DY1750), NGAL (R&D Systems^®^: cat# DY1757), e Syndecan-1 (Abcam^®^ ab47352). Os procedimentos seguiram as recomendações do fabricante e estão representados no diagrama gráfico (Figura 1).

**Figura 1:** Medições de biomarcadores renais e endoteliais. Notas: Imagens obtidas de fontes públicas e sob licença Creative Commons: [1] https://images.app.goo.gl/hVp4Kq5j3sosjRiX7; [2] https://images.app.goo.gl/z2RinjcSy1JAVZFb9; [3] https://images.app.goo.gl/U5FMjAxiBUx8S2ok6; [4] https://commons.wikimedia.org/w/index.php?curid=12484442; [5] https://commons.wikimedia.org/wiki/File:Blausen_0468_Heart-Lung_Machine.png; [6] https://commons.wikimedia.org/w/index.php?curid=4489929.

### Análise Estatística

As variáveis categóricas foram expressas como números absolutos e porcentagens. As variáveis quantitativas foram testadas quanto à distribuição normal por meio do teste de Shapiro-Wilk. A assimetria e a curtose dos dados foram analisadas por meio de histogramas e gráficos Q-Q. Os dados normais foram expressos como média ± desvio padrão e os dados não normais como mediana e intervalo interquartil. Para comparações entre dados categóricos, foram usados os testes qui-quadrado e exato de Fisher, conforme apropriado. Na análise pareada com comparações entre grupos dependentes de acordo com os períodos de cirurgia, foi utilizado o teste de Friedman para dados não normais. Em caso de significância estatística, o teste de Wilcoxon foi utilizado para comparações pareadas, em que o valor crítico de significância estatística foi dividido pelo número de grupos pareados (três períodos): para considerar comparações múltiplas e reduzir o risco de erro do tipo I, foi aplicado um valor p limite de 0,016 para significância estatística. Para os demais testes, p < 0,05 foi considerado estatisticamente significativo. Os dados foram analisados usando o software SPSS para Macintosh, versão 23 (Armonk, NY: IBM Corp).

## Resultados

Um total de 30 pacientes foram inscritos, porém 8 foram excluídos por diversos motivos: perda de acompanhamento, diagnóstico tardio de insuficiência renal crônica e recusa em continuar com o registro de informações para a pesquisa. Dessa forma, 22 pacientes permaneceram no estudo. A idade média foi de 65 ± 8 anos e 68% eram do sexo masculino. Hipertensão arterial (91%) e diabetes (36%) foram comorbidades altamente prevalentes. O tabagismo esteve presente em 23%, e o histórico familiar de DAC foi observado na mesma porcentagem de pacientes. O escore Syntax mais frequente foi 3 (91%) (Tabela 1).

**Tabela 1:** Aspectos clínicos e cirúrgicos e hospitalização pré-operatória dos pacientes incluídos

	Grupo total (n = 22)
Aspectos clínicos	
Idade, anos	65 ± 8
Masculino, sexo	15 (68,2)
Etnia	
Branca	3 (13,6)
Parda	19 (86,4)
Comorbidades e fatores de risco	
Hipertensão arterial	20 (90,9)
Diabetes	8 (36,4)
Tabagismo	5 (22,7)
Coronopatia de histórico familiar	5 (22,7)
Obesidade	1 (4,5)
Dislipidemia	11 (50)
Outros	7 (31,8)
Escore Syntax	
1	1 (4,5)
2	1 (4,5)
3	19 (91)
Escore STS	0,92 (0,58–2,58)
FEVEs	52 ± 8
Aspectos cirúrgicos e hospitalização	
CEC	
Não	13 (59,1)
Sim	9 (40,9)
Tempo de CEC (min)	86 ± 27
Hipotermia	6 (27,7)
Tempo de permanência hospitalar, dias	9 (5–12)
Tempo entre a cirurgia e a alta hospitalar, dias	7 (5–10)

Abreviações – CEC: circulação extracorpórea. Notas – Dados quantitativos expressos como média ± desvio padrão ou mediana e intervalo interquartil entre parênteses. Dados categóricos expressos como contagens absolutas e porcentagens entre parênteses.

Com relação aos aspectos cirúrgicos, nove pacientes (41%) foram submetidos ao procedimento de CEC. A duração média da CEC foi de 86 ± 27 minutos. A mediana com intervalo interquartil (IIQ) para o tempo de permanência hospitalar foi de 9 (5–12) dias, e o tempo entre a cirurgia e a alta hospitalar foi de 7 (5–10) dias (Tabela 1). Não houve diferença estatística em nenhum dos aspectos clínicos nas comparações independentes entre os grupos. O escore STS foi maior (2,52 [IIQ: 0,92–3,03] vs. 0,58 [IIQ: 0,31–0,81], p = 0,030) e a FEVE foi menor (47 ± 7 vs. 59 ± 2, p = 0,013) no grupo *on-pump*. O grupo *on-pump* apresentou maior frequência de hipotermia induzida (75% vs. 0%; p = 0,001), pois a redução da temperatura corporal faz parte da técnica de revascularização. Não houve diferenças estatísticas no tempo entre a cirurgia e a alta hospitalar, nem nos dados laboratoriais coletados antes da cirurgia (Tabela 2).

**Tabela 2:** Aspectos clínicos, cirúrgicos e hospitalização dos pacientes incluídos de acordo com o uso de *bypass* da artéria coronária durante a cirurgia

	Sem CEC (n = 13)	CEC (n = 9)	p*
Aspectos clínicos			
Idade, anos	63 ± 7	68 ± 9	0,176
Masculino, sexo	9 (69,2)	6 (66,7)	1,000
Etnia			1,000
Branca	2 (15,4)	1 (11,1)	
Parda	11 (84,6)	8 (88,9)	
Comorbidades e fatores de risco			
Hipertensão arterial	12 (92,3)	8 (88,9)	1,000
Diabetes	7 (53,8)	1 (11,1)	0,074
Tabagismo	4 (30,8)	1 (11,1)	0,360
DAOP	0 (0)	0 (0)	–
Arritmias	0 (0)	0 (0)	–
Coronopatia de histórico familiar	2 (15,4)	3 (33,3)	0,609
Obesidade	0 (0)	1 (11,1)	0,409
Dislipidemia	6 (46,2)	5 (55,6)	1,000
Outros	5 (38,5)	2 (22,2)	0,648
Escore Syntax			0,114
1	0 (0)	1 (11,1)	
2	0 (0)	1 (11,1)	
3	12 (100)	7 (77,8)	
Escore STS	0,58 (0,31–0,81)	2,52 (0,92–3,03)	0,030
FEVEs	59 ± 2	47 ± 7	0,013
Hipotermia e permanência hospitalar			
Hipotermia	0 (0)	6 (75)	0,001
Tempo de permanência hospitalar, dias	8 (7–12)	10 (5–11)	0,896
Tempo entre a cirurgia e a alta hospitalar, dias	7 (6–11)	7 (5–8)	0,744
Laboratoriais (antes da cirurgia)			
Hemoglobina (g/dL)	13,2 ± 1,9	12,7 ± 2	0,560
Hematócritos (%)	40 ± 5	38 ± 6	0,346
Sódio (mEq/L)	139 ± 3	142 ± 8	0,291
Potássio (mEq/L)	4,61 ± 0,41	4,38 ± 0,29	0,180
Leucócitos	7596 (6200–8760)	8240 (7673–12800)	0,169
Plaquetas	248833 ± 38442	211711 ± 64056	0,114
Ureia sérica (mg/dL)	42 ± 10	43 ± 18	0,780
Creatinina (mg/dL)	0,84 (0,75–1,05)	1 (0,8–1,1)	0,651
TFGe (mL/min.1,73m^2^)	89 ± 21	76 ± 30	0,266

Abreviações – CEC: circulação extracorpórea. TFGe: taxa de filtração glomerular estimada. Notas – Dados quantitativos expressos como média ± desvio padrão ou como mediana e intervalo interquartil entre parênteses. Dados categóricos expressos como contagens absolutas e porcentagens entre parênteses.

*Para dados categóricos, utilizou-se o teste exato de Fisher ou o teste qui-quadrado. Para dados quantitativos, foram utilizados o teste t de Student para dados normais e o teste de Mann-Whitney para dados não normais.

Com relação aos novos biomarcadores, durante a CRM, o grupo CEC apresentou níveis aumentados de nefrina urinária em comparação ao grupo *off-pump* (2.051,42 [1.018,82–4.166,25] vs. 400 [27,59–1.186,9] pg/mg-Cr; p = 0,007). Além disso, os níveis de NGAL urinário e sérico foram mais elevados no grupo *on-pump* em comparação ao grupo *off-pump* (Tabela 2). O mesmo efeito foi observado para a NGAL na urina (24 [13,28–31,58] vs. 7,24 [4,55–14,18] ng/mg-Cr; p = 0,036) e no soro (282,12 [232,5–312,25] vs. 165,15 [126,16–186,42] ng/mL; p = 0,030). Não houve diferenças estatísticas em relação aos demais parâmetros cirúrgicos (Tabela 3). Ademais, nas comparações pareadas, a nefrina urinária, a NGAL urinária e a NGAL sérica aumentaram significativamente durante a cirurgia somente no grupo *on-pump*. Esses níveis permaneceram elevados mesmo após o procedimento, ou seja, na admissão na UTI, em comparação aos valores “pré-cirurgia” (Tabela 4 e Figura 2).

**Tabela 3:** Comparação dos níveis de biomarcadores em cada período cirúrgico em pacientes submetidos ou não à circulação extracorpórea (CEC) durante a cirurgia

	Sem CEC (n = 13)	CEC (n = 9)	p*
Antes da cirurgia			
uNefrina (pg/mg-Cr)	341,73 (33,33–1654,98)	887,56 (381,4–1168,28)	0,556
uMCP-1 (pg/mg-Cr)	35 (10,09–56,88)	13,62 (1,7–129,7)	0,794
uKIM-1 (pg/mg-Cr)	3860 (842–8245)	9651 (464–66589)	0,473
uNGAL (ng/mg-Cr)	5,17 (2,5–6,51)	2,86 (0,84–7,76)	0,431
sNGAL (ng/mL)	90,72 (74,77–136,79)	142,11 (117,3–234,27)	0,393
Syndecan-1 (ng/mL)	75,21 (68,5–99,49)	75,85 (68,18–86,39)	0,601
Durante a cirurgia			
uNefrina (pg/mg-Cr)	400 (27,59–1186,9)	2051,42 (1018,82–4166,25)	**0,007**
uMCP-1 (pg/mg-Cr)	16,08 (2,33–154,65)	4,25 (2,01–47,65)	0,492
uKIM-1 (pg/mg-Cr)	2616 (966–13506)	7330 (2978–28377)	0,460
uNGAL (ng/mg-Cr)	7,24 (4,55–14,18)	24 (13,28–31,58)	**0,036**
sNGAL (ng/mL)	165,15 (126,16–186,42)	282,12 (232,5–312,25)	**0,030**
Syndecan-1 (ng/mL)	126,34 (93,42–367,92)	116,11 (77,44–287,08)	0,393
Após a cirurgia (admissão na UTI)			
uNefrina (pg/mg-Cr)	1054,84 (190,05–3384,75)	1806,52 (958,33–3142,08)	0,395
uMCP-1 (pg/mg-Cr)	2,39 (1,21–35,75)	2,03 (0,94–46,58)	0,762
uKIM-1 (pg/mg-Cr)	4242 (20–41253)	1584 (254–21520)	0,840
uNGAL (ng/mg-Cr)	7,51 (5,37–18,18)	17,38 (4,57–30,15)	0,431
sNGAL (ng/mL)	189,96 (129,71–253,76)	207,68 (186,42–324,65)	0,431
Syndecan-1 (ng/mL)	88,31 (66,58–227,96)	80,96 (73,93–161,49)	0,984

Abreviações – CEC: circulação extracorpórea. Notas – Dados quantitativos expressos como mediana e intervalo interquartil entre parênteses.

*Foi utilizado o teste de Mann-Whitney.

**Tabela 4:** Comparação pareada dos níveis de biomarcadores entre cada período do estudo em pacientes submetidos ou não à circulação extracorpórea

	Períodos			p*
	Antes da cirurgia	Durante a cirurgia	Após a cirurgia (admissão na UTI)	
Grupo CEC				
uNefrina (pg/mg-Cr)	887,56 (381,4–1168,28)	2051,42 (1018,82–4166,25)	1806,52 (958,33–3142,08)	**0,002A**
uMCP-1 (pg/mg-Cr)	13,62 (1,7–129,7)	4,25 (2,01–47,65)	2,03 (0,94–46,58)	0,135
uKIM-1 (pg/mg-Cr)	9651 (464–66589)	7330 (2978–28377)	1584 (254–21520)	0,867
uNGAL (ng/mg-Cr)	2,86 (0,84–7,76)	24 (13,28–31,58)	17,38 (4,57–30,15)	**0,001A**
sNGAL (ng/mL)	142,11 (117,3–234,27)	282,12 (232,5–312,25)	207,68 (186,42–324,65)	**0,001B**
Syndecan-1 (ng/mL)	75,85 (68,18–86,39)	116,11 (77,44–287,08)	80,96 (73,93–161,49)	0,717
Pacientes sem CEC				
uNefrina (pg/mg-Cr)	341,73 (33,33–1654,98)	400 (27,59–1186,9)	1054,84 (190,05–3384,75)	0,092
uMCP-1 (pg/mg-Cr)	35 (10,09–56,88)	16,08 (2,33–154,65)	2,39 (1,21–35,75)	0,459
uKIM-1 (pg/mg-Cr)	3860 (842–8245)	2616 (966–13506)	4242 (20–41253)	0,717
uNGAL (ng/mg-Cr)	5,17 (2,5–6,51)	7,24 (4,55–14,18)	7,51 (5,37–18,18)	0,500
sNGAL (ng/mL)	90,72 (74,77–136,79)	165,15 (126,16–186,42)	189,96 (129,71–253,76)	0,063
Syndecan-1 (ng/mL)	75,21 (68,5–99,49)	126,34 (93,42–367,92)	88,31 (66,58–227,96)	0,092

Abreviações – CEC: circulação extracorpórea. Notas – Dados quantitativos expressos como mediana e intervalo interquartil entre parênteses.

*Foi utilizado o teste de Friedman. A: Significância observada entre “Antes da cirurgia” vs. outros períodos. B: Significância observada entre todos os períodos.

**Figura 2:** A NGAL urinária, a nefrina urinária e a NGAL sérica aumentaram significativamente durante a cirurgia no grupo *on-pump*.

Por fim, o aumento dos níveis urinários de NGAL durante a cirurgia em pacientes submetidos à CEC (*on-pump*) correlacionou-se com a redução da TFG nas primeiras 48 horas após a cirurgia cardíaca (Rho = −0,838; p = 0,009). No entanto, não foi observada nenhuma correlação significativa no grupo *off-pump* (Figura 3).

**Figura 3:** Correlação entre o nível de NGAL urinária durante a CRM e a variação da TFG nas primeiras 48 horas após a cirurgia.

## Discussão

No presente estudo, demonstramos que a CEC utilizada durante a cirurgia de revascularização do miocárdio esteve associada a danos às estruturas glomerulares e tubulares do rim e a uma redução adicional na TFG 48 horas após a cirurgia. Os biomarcadores urinários foram úteis para demonstrar que o uso de CEC na CRM *on-pump* foi associado ao desenvolvimento de doença renal posterior em comparação à cirurgia *off-pump*, devendo esses pacientes ser monitorados no período pós-operatório para evitar complicações renais.

A patogênese da IRA pós-operatória é multifatorial e envolve diversos mecanismos, como hipoperfusão renal, lesão por isquemia e reperfusão, ativação neuro-humoral, inflamação, estresse oxidativo, nefrotoxinas e fatores mecânicos^10,16^. Além disso, pacientes com disfunção renal prévia apresentam maior risco de desenvolver IRA após a cirurgia, o que está associado a mau prognóstico e óbito^9^. O diagnóstico precoce e preciso da disfunção renal pode melhorar a estratificação do risco de IRA após a cirurgia de revascularização do miocárdio e permitir estratégias adequadas de prevenção e tratamento^3^. Em uma revisão sistemática de 33 estudos controlados e randomizados com mais de 1.700 pacientes submetidos a CRM, os indivíduos que passaram por procedimentos *off-pump* foram associados a um risco reduzido de IRA. Apesar disso, o estudo ROOBY, com 2203 pacientes, não encontrou nenhum benefício de sobrevida ou efeito renoprotetor em pacientes submetidos à revascularização *off-pump* em comparação àqueles que utilizaram assistência circulatória com *bypass*, incluindo uma taxa mais elevada de morte cardíaca e menor patência do enxerto no primeiro ano de acompanhamento. Especulamos que o procedimento de CEC pode potencialmente causar um dano importante na estrutura renal, levando à redução aguda da TFG^17^. Em um estudo recente com pacientes com ICFER em estado agudo descompensado submetidos à CRM *on-pump*, Simão et al.^18^ demonstraram uma resposta inflamatória e sugeriram que a exacerbação do estado pró-inflamatório secundário à doença aguda e à CEC exerceu um efeito sinérgico devastador, que contribuiu para o óbito dos pacientes do grupo *on-pump*.

No presente estudo, os pacientes submetidos à CRM *on-pump* apresentaram níveis aumentados de NGAL urinária e sérica em comparação à CRM *off-pump*, principalmente no período intraoperatório. Além disso, os níveis de NGAL urinária durante o período intraoperatório da CRM com suporte de CEC foram associados a uma redução adicional da TFG. A NGAL é tipicamente uma proteína secretora de baixo peso molecular, originalmente identificada em neutrófilos. Posteriormente, descobriu-se que ela está presente em outros órgãos, incluindo os rins^19,20^. Estudos identificaram a NGAL como um dos genes mais surpreendentemente regulados (e proteínas superexpressas no rim após isquemia)^21^.

Os primeiros estudos com a NGAL como biomarcador precoce de IRA em amostras biológicas foram realizados no contexto de cirurgias cardíacas^22^. Na sequência, foram realizados diversos estudos para investigar o desempenho diagnóstico da NGAL urinária e sanguínea para IRA. Um escore cardíaco baseado nos valores de NGAL associados à cirurgia chegou a ser proposto e também utilizado em um algoritmo de decisão para modificações no procedimento cirúrgico, incluindo o reagendamento da cirurgia^11^.

Em uma meta-análise envolvendo 16 estudos que avaliaram a utilidade da NGAL para o diagnóstico de IRA após cirurgia cardíaca com necessidade de CEC, o valor diagnóstico da NGAL plasmática foi inconclusivo, exigindo investigações adicionais^23^. O desempenho diagnóstico da NGAL avaliada de 4 a 8 horas após a interrupção da CEC em pacientes de cirurgia cardíaca foi superior à NGAL avaliada em menos de 4 horas e em 24 horas para o diagnóstico precoce de IRA. Apesar do papel potencial para a utilidade diagnóstica da NGAL nesse cenário clínico, existem poucos estudos, com heterogeneidade substancial e conclusões fracas^23^. Assim, o presente estudo se mostra relevante ao distinguir o envolvimento da NGAL em um tipo específico de cirurgia cardíaca (revascularização do miocárdio – CRM) com CEC em comparação à cirurgia *off-pump*, comprovando que as alterações nos níveis de NGAL, tanto na urina quanto no sangue, estiveram associadas ao procedimento cirúrgico com CEC.

Este estudo demonstrou pela primeira vez que os níveis de nefrina urinária ficam elevados durante o período intraoperatório da cirurgia cardíaca, especialmente no grupo *on-pump*. A nefrina é a primeira proteína transmembrana de globulina localizada no diafragma de fenda de filtração, que desempenha um papel fundamental nos pedicelos dos podócitos relacionados à permeabilidade seletiva da barreira de filtração glomerular, evitando a ligação de proteínas^24^. Consequentemente, de forma pioneira, demonstra que essa proteína atua como um marcador precoce de injúria podocitária na IRA mediada por CEC. Estudos experimentais demonstraram que as alterações na expressão da nefrina foram associadas à hipertensão e à resposta ao tratamento anti-hipertensivo^25^. Nas células, a expressão reduzida da nefrina tem sido relatada como indicativa de lesão nos podócitos, o que causa o rompimento dos processos podais e do diafragma de fenda^26^. Como consequência, a nefrinúria foi associada ao descolamento dos podócitos, levando à podocitopenia e, em seguida, à glomeruloesclerose^27^. Em outro estudo realizado com pacientes diabéticos, divididos de acordo com os níveis de albuminúria, os níveis de nefrina urinária foram correlacionados negativamente com a TFG, indicando que a nefrinúria é um marcador de disfunção renal^28^. No mesmo estudo, sugeriu-se que a nefrinúria pode ser mais útil como biomarcador precoce de DRC do que a albuminúria.

Até onde temos conhecimento, não há estudos que avaliem os níveis de nefrina urinária no contexto de cirurgias cardíacas em humanos. Em um modelo experimental de injúria renal aguda por isquemia-reperfusão, a injúria podocitária foi um alvo importante e a injúria por isquemia-reperfusão de 30 minutos foi associada à redução da expressão de nefrina nos podócitos, ao aumento da proteinúria, ao declínio da função renal e ao aumento dos marcadores de fibrose, que contribuem para a disfunção permanente das células renais^29^.

Na presente pesquisa, os níveis elevados de nefrina podem refletir injúria podocitária não apenas em cirurgias cardíacas, mas especificamente na CRM sem o procedimento *on-pump*. Esses níveis devem ser monitorados com o objetivo de investigar outras complicações renais relacionadas aos mecanismos de fibrose, como na DRC.

Os níveis urinários de MCP-1 avaliados neste estudo na admissão à UTI após cirurgia cardíaca correlacionaram-se significativamente com a diminuição da TFG nas primeiras 48 horas no grupo CEC. O MCP-1 urinário é um biomarcador bem reconhecido de DRC e tem sido associado a mecanismos de fibrose^30,31^. Além disso, o MCP-1 parece contribuir para a inflamação renal e alterações na expressão de nefrina, levando à injúria podocitária e a manifestações clínicas como proteinúria, albuminúria e TFG reduzida^32,33^. Assim, especulamos que a injúria por isquemia-reperfusão induzida pela CRM *on-pump* possa ativar mecanismos inflamatórios e de injúria podocitária que persistem após a cirurgia e podem contribuir para a injúria renal e a redução da TFG nas primeiras 48 horas.

No contexto internacional, as taxas de prevalência e incidência de DRC vêm aumentando, principalmente entre adultos com mais de 30 anos de idade, segundo a Sociedade Brasileira de Nefrologia. Considerando que o tratamento da DRC é oneroso e sobrecarrega os sistemas de saúde, o diagnóstico precoce e correto da IRA reveste-se de importância social pela adoção de medidas terapêuticas em tempo hábil ou pela correção de causas potencialmente reversíveis, oferecendo oportunidades de prevenção ou limitando a progressão do dano renal para uma DRC que demande suporte especializado. Nesse sentido, biomarcadores não convencionais, como NGAL, MCP-1, KIM-1 e nefrina, são descritos em estudos recentes como promissores para a avaliação precoce de IRA devido à sua elevada sensibilidade, demonstrando um aumento significativo em vários contextos clínicos, mesmo antes da manifestação dos sinais e sintomas clássicos de IRA^34^.

Pesquisas anteriores destacaram a importância do uso de biomarcadores não convencionais para o diagnóstico precoce de IRA em recém-nascidos prematuros diagnosticados com sepse, contribuindo para a tomada de decisões mais assertiva e evitando o manejo terapêutico tardio desses pacientes^35^. Além disso, uma vez que a COVID-19 constitui uma infecção pró-inflamatória e sistêmica, a IRA se manifesta como uma complicação que agrava o quadro clínico do paciente, o que pode resultar em perda de função e óbito, e o uso de biomarcadores, especialmente a NGAL urinária, tem se mostrado útil na predição do prognóstico de pacientes infectados^36^. A função renal foi correlacionada com a esquistossomose hepatoesplênica (EHE), indicando que a disfunção renal está relacionada à EHE, principalmente a disfunção tubular, verificada pela concentração urinária e acidificação distal incompleta, sendo a MCP-1 urinária um biomarcador mais sensível do que a taxa de excreção de albumina urinária^37^.

Este estudo teve limitações. Foi um estudo unicêntrico, com um tamanho de amostra pequeno, o que dificultou a análise de regressão. Além disso, não foram avaliados outros biomarcadores urinários, como a proteinúria e a albuminúria, que poderiam ajudar a avaliar as alterações na estrutura glomerular. O uso de CEC durante a cirurgia cardíaca foi associado a danos renais estruturais, incluindo injúria tubular e podocitária, bem como injúria renal de curto prazo. Além disso, a análise estatística foi principalmente univariada, o que nos impediu de atribuir conclusivamente o aumento dos níveis de biomarcadores somente à CEC em uma pequena amostra de nove pacientes. Outros fatores perioperatórios podem ter influenciado as associações observadas. Adicionalmente, a determinação do tamanho amostral necessário para confirmar a magnitude do efeito da CEC exigiria uma análise formal do poder. Estudos futuros com coortes maiores e análise multivariada são necessários para isolar de forma mais adequada o impacto independente da CEC nas alterações dos biomarcadores.

Em conclusão, novos biomarcadores podem ser úteis na detecção precoce da DRC, na qual a NGAL urinária foi capaz de antecipar um comprometimento da filtração glomerular mediado pela CRM em 48 horas.

## Data Availability

Os dados utilizados neste estudo não estão disponíveis publicamente devido a acordos de confidencialidade, conforme estipulado pelo comitê de ética, pelo consentimento dos participantes ou pela instituição responsável. O acesso aos dados pode ser considerado mediante solicitação formal e aprovação prévia das partes relevantes, de acordo com os requisitos de confidencialidade e a proteção das informações sensíveis.

## Material Suplementar

O seguinte material online está disponível para este artigo:

Tabela S1 – Análise de correlação entre biomarcadores e variação da taxa de filtração glomerular nas primeiras 48 horas após a cirurgia.
